# Videonystagmography features and clinical symptoms correlate with Parkinson's clinical subtypes

**DOI:** 10.3389/fneur.2025.1693803

**Published:** 2025-11-26

**Authors:** Guangli Zheng, Ruike Xie, Yuanyuan Ma, Zhibin Chen, Tan Wang, Ye Xu

**Affiliations:** 1 Department of Neurology, The First Affiliated Hospital of Hainan Medical University, Haikou, China; 2 Department of Neurology, The Danzhou People's Hospital of Hainan, Danzhou, China

**Keywords:** Parkinson's disease, videonystagmography, motor symptoms, non-motor symptoms, correlation analysis

## Abstract

**Objective:**

Analyzing the differences in video-nystagmography (VNG) parameters among Parkinson's disease (PD) patients with distinct motor subtypes and evaluate the correlation between VNG parameters and the severity of non-motor symptoms (NMS) in Tremer Dominant (TD)/Postural Instability/Gait Difficulty (PIGD) subtypes provides a theoretical basis for advancing the understanding of PD heterogeneity.

**Methods:**

Sixty-nine patients with PD diagnosed in the Department of Neurology of the First Affiliated Hospital of Hainan Medical College from December 2022 to January 2024 were collected. Among them, 35 were in the Tremer Dominant (TD) group, 34 were in the Postural Instability/Gait Difficulty (PIGD) group, and 38 patients with physical examination admitted in the same period were collected as the healthy control (HC) group. The subjects were assessed for motor and non-motor symptoms, and the VNG was refined to record the assessment of eye movement abnormalities in the patients. The general clinical data and VNG parameters of the subjects in the three groups were analyzed and correlation analyses were performed for clinical features and nystagmus views with intergroup differences in the PIGD and TD groups.

**Results:**

There were significant differences in VNG parameters among the three groups of subjects, mainly in vertical sweep latency and solid-phase microextraction (SPEM) vertical gain (*P* < 0.05). The differences between the TD group and the PIGD group were statistically significant in terms of Hoehn–Yahr (H–Y) classification (*P* = 0.013), Movement Disorder Society-Unified Parkinson's Disease Rating Scale (MDS-UPDRS) III (*P* = 0.010), Hamilton Anxiety Scale (HAMA; *P* = 0.024), and Hamilton Depression Scale (HAMD; *P* = 0.021). There were no significant differences in MDS-UPDRS I (*P* = 0.751), MDS-UPDRS II (*P* = 0.088), Mini-Mental State Examination (MMSE; *P* = 0.413) and Montreal Cognitive Assessment (MoCa; *P* = 0.341) scores. Correlation analysis showed that VNG in the PIGD group was negatively correlated with the MDS-UPDRS III score and H-Y grading, correlated with the HAMD score (*P* < 0.05), and not correlated with the HAMA score (*P* > 0.05). In the TD patients, VNG was negatively correlated with the HAMA and HAMD scores (*P* < 0.05), and was not correlated with the MDS-UPDRS III score and H-Y grading (*P* > 0.05).

**Conclusion:**

VNG shows significant differences among Parkinson's disease patients with different motor subtypes, and VNG features are correlated with non-motor symptoms.

## Introduction

Parkinson's disease (PD) is a common chronic degenerative disease of the central nervous system in middle-aged and elderly people, with a prevalence of about 17 cases per 1,000 people over the age of 65 in China, and its prevalence continues to increase with age (1). Clinically, it is mainly characterized by motor symptoms (MS) such as motor retardation, resting tremor, and postural coordination disorder, and non-motor symptoms (NMS) such as cognitive impairment and autonomic dysfunction (2). It has been found that NMS predominates in the early stages of PD, with MS appearing in the later stages, when a lengthy degeneration of the nervous system has occurred. Based on the MS of the patients, they are classified into (Tremer Dominant) TD predominant, Postural Instability/Gait Difficulty (PIGD) predominant and indeterminate subtypes characterized by tremor, postural gait abnormality and mixed type, respectively (3). Evidence suggests that there are significant differences in terms of NMS in patients with different PD motor subtypes, with patients with PIGD being more likely to have concomitant NMS such as cognitive deficits, anxiety, and a poorer prognosis compared to other subtypes (4–6). Therefore, the accurate differentiation of PD patient subtypes is a crucial measure for improving patient prognosis.

Evidence indicates that eye movements and gait/postural control share overlapping neural networks, primarily manifested in visually guided movements. Eye movements and gait control jointly regulate locomotion, with these regulatory mechanisms predominantly mediated by the cortico-basal ganglia-brainstem-cerebellar pathway (7). The basal nuclei are intermediate circuits that regulate eye movements, leading to the hypothesis that PD patients may also have oculomotor deficits that may occur after or before motor symptoms. Since 1983 there has been increasing evidence of oculomotor abnormalities in PD patients, which may affect balance and posture/gait in PD patients (8, 9). A small number of studies have revealed that patients with PIGD have increased sweep latencies and that PD patients with frozen gait have worse postural control (10, 11). This suggests that different clinical phenotypes may correspond to distinct patterns of neural circuit damage. Therefore, exploring oculomotor abnormalities in PD patients may provide significant value in the early diagnosis, progression, and differential diagnosis of PD. Dopaminergic agents, as anti-Parkinson's disease medications, improve the function of relevant neural circuits by supplementing or mimicking dopamine. This ameliorative effect manifests through their action on the basal ganglia-thalamic-cortical circuit, simultaneously enhancing motor initiation and execution functions in both eye movements and gait (12). It has been found that some patients with levodopa or dopaminergic medication have improved swept eye movements and solid-phase microextraction (SPEM) (13). Ocular neurological impairment is attributed to dopamine depletion, but there are still different opinions regarding the role of dopamine therapy in correcting oculomotor dysfunction (14). Furthermore, a meta-analysis of studies still lacks conclusive evidence demonstrating a direct association between ocular motor abnormalities and the dopaminergic system (15, 16). Video-nystagmography (VNG) serves as a neurophysiological technique for objectively assessing vestibulo-ocular system function. It demonstrates predictive value for changes in ocular motor function in PD patients (17), with specific indicators providing objective neurophysiological evidence to a certain extent. The VNG can make the study of the vestibular labyrinth and its connections and pathways to the central nervous system more sensitive, and accomplishes both peripheral and local diagnosis of central vestibular loss (18). Quantitative analysis of subjects' eye movement patterns by recording their eye movements under different conditions provides a non-invasive and stable assessment index for PD patients. Therefore, the VNG parameters more directly represents ocular motility abnormalities in PD patients.

Based on the advisory research of the MSD Subtype Classification Working Group and guided by its principles of pursuing biological significance, emphasizing clinical utility, advocating objectivity and standardization, and addressing heterogeneity (19), this study investigated the correlation between the potential objective biomarker VNG parameters and PD motor subtypes and non-motor symptoms. This provides a theoretical foundation for further understanding the heterogeneity of PD subtypes.

## Materials and methods

### Study design

This study is a case-control investigation aimed at exploring the correlation between VNG and different subtypes of Parkinson's disease, thereby providing improved clinical tools for distinguishing between distinct subtypes of PD patients.

### Participants

Sixty-nine PD patients and 38 healthy people diagnosed in the Department of Neurology of the First Affiliated Hospital of Hainan Medical College from December 2022 to January 2024 were selected as the study subjects. Inclusion criteria of PD patients: met the Chinese diagnostic criteria for Parkinson's disease (2016 edition). This edition builds upon the 2015 MSD criteria, incorporating adjustments and refinements tailored to domestic circumstances. Both versions maintain high consistency in core diagnostic foundations, diagnostic tier classification, and fundamental logical frameworks. The principal distinctions are: (1) the 2016 Chinese criteria explicitly designate three supplementary tests as “supporting criteria”: hyposmia or anosmia; cranial ultrasound demonstrating abnormal hyper-echogenicity in the substantia nigra (>20 mm^2^); and cardiac MIBG scintigraphy revealing cardiac denervation. (2) The 2016 Chinese criteria provide clearer quantitative standards for the offsetting rules of warning signs. (3) The 2016 Chinese criteria place greater emphasis on considerations for early diagnosis. Exclusion criteria of PD patients: ① secondary Parkinson's disease due to pharmacological, traumatic, toxic, and vascular causes; ② dementia with Lewy bodies, progressive supranuclear palsy, and multiple system atrophy are examples of Parkinson's-plus syndromes; ③ those with Parkinson's disease dementia or severe motor deficits who are unable to cooperate in completing the nystagmus view examination; ④ patients with psychiatric symptoms such as severe anxiety and depression; ⑤ ocular diseases such as glaucoma, high myopia, ocular trauma, or history of fundus laser treatment; ⑥ Patients may present with blepharospasm, diplopia, ophthalmoplegia, hearing loss, dizziness, tinnitus, corticobasal syndrome, normal pressure hydrocephalus, non-motor fluctuations, foot deformities, orthopedic complications, and other conditions. Healthy control (HC) patients inclusion criteria: medical examination patients whose admission age and sex were matched to the PD group and who voluntarily underwent nystagmus view examination. The study was approved by the Hospital Ethics Committee (2023-KYL-217) and all subjects signed an informed consent form before enrolment.

### Assessment of clinical features

MS was scored using the Movement Disorder Society-Unified Parkinson's Disease Rating Scale (MDS-UPDRS) Part III; the severity of the disease was graded using the Hoehn-Yahr (H-Y) scale. The H-Y scale grades 1–2 were for early stage, 2.5–3 for intermediate stage, and 4–5 for advanced stage of the disease. The MDS-UPDRS Scale Part I and MDS-UPDRS Scale Part II were used to assess daily living activities and NMS. The Hamilton Depression Scale (HAMD) and Hamilton Anxiety Scale (HAMA) were used to assess the mental state of PD patients, and the Montreal Cognitive Assessment (MoCa) scale and Mini-Mental State Examination (MMSE) scale was used to assess the cognitive function of patients with PD. All scales were completed in the OFF state and rated separately by two trained clinicians, and the results were averaged.

According to the MDS-UPDRS III score (20), PD patients were classified into the tremor subtype and the postural gait abnormality subtype. The specific criteria were as follows (21, 22): the mean values of the tremor items including MDS-UPDRS 2.10, 3.15a, 3.15b, 3.16a, 3.16b, 3.17a, 3.17b, 3.17c, 3.17d, 3.17e, and 3.18 were calculated based on the MDS-UPDRS scores, respectively, divided by the mean values of the symptomatic items of abnormal postural gait including MDS The mean scores of -UPDRS 2.12, 2.13, 3.10, 3.11, and 3.12 are obtained as the ratio of the mean score of the tremor items to the mean score of the postural gait abnormality items, and if the resulting ratio is greater than or equal to 1.15, then the patient is classified as TD. If the ratio is less than or equal to 0.90, then the patient is classified as a PIGD. Patients with ratios between 0.9 and 1.15 were classified as intermediate type; however, in this cohort, no patients fell within this intermediate range. Consequently, all participants were definitively categorized as either TD or PIGD. If the tremor score is not 0 and the Postural Abnormal Gait Disorder score was 0, then the patient was classified as TD-PD; if the tremor score was 0 and the Postural Abnormal Gait Disorder score was not 0, then the patient was classified as PIGD-PD.

### VNG data acquisition and preprocessing

The ZT-VNG-I Benign Paroxysmal Positional Vertigo Diagnostic Instrument, manufactured by Tianjin Zhiting Medical Technology Co., Ltd., was employed to conduct ocular motility examinations on subjects. The entire testing procedure was conducted in a quiet, darkened environment. Prior to the test tasks, bedside assessments of ocular motility were performed on all subjects to observe the speed, direction, amplitude, and smoothness of eye movements. Ocular movement trajectories were recorded on a horizontal plane to evaluate eye movement tasks. The computer system identified and processed data based on the characteristics of the nystagmogram waveform, displaying the waveform on the monitor. Simultaneously, based on changes in the position of ocular movement, the computer calculated movement parameters such as the saccade latency, saccade accuracy, and SPEM gain at different frequencies. These movement parameter data served as diagnostic references. The saccade latency refers to the time interval from the appearance of a visual target to the initiation of ocular movement toward that target, reflecting the reaction speed in initiating saccadic actions. Saccadic accuracy denotes the proximity of the final fixation point to the target location, indicating execution deficits in ocular movements that may correlate with gait disturbance mechanisms. SPEM gain serves as an indicator of the eye's ability to smoothly track moving targets, utilized to assess cortical function and disease severity. During test result analysis, the examining clinician excludes ocular activity data caused by blinking, inadvertent head movements, and measurement errors to prevent interference with statistical outcomes. All electroretinography examinations are conducted by two neurologists specializing in PD diagnosis and treatment, both trained in electroretinography. The specific data collection process is detailed in the Supplementary material.

### Statistical analysis

Statistical analysis of the data was performed using SPSS 26.0. For quantitative data, normality was assessed, data meeting normality requirements were expressed as mean ± standard deviation. Comparisons between two groups employed the independent samples *t*-test, while comparisons among multiple groups utilized one-way analysis of variance (ANOVA), with pairwise comparisons conducted using the LSD method. Data not meeting normality requirements were expressed as median (interquartile range). Comparisons between two groups employed the Mann–Whitney *U* test, while multiple group comparisons utilized the Kruskal–Wallis *H* test. Pairwise comparisons were conducted using the Nemenyi method. Categorical count data were presented as case numbers (%). Group comparisons were performed using the chi-square (χ^2^) test. Spearman's correlation analysis was employed to assess relationships between variables. Given multiple comparisons within the correlation analysis, *P*-values underwent Benjamini–Hochberg correction to control the false discovery rate at 5%. The report presents the adjusted *P*-values. *P* < 0.05 was considered statistically significant.

## Results

### Demographic and clinical characteristics

There was no statistically significant difference between the three groups in terms of gender, age and education (*P* > 0.05), and there was no significant difference between PIGD and TD patients in terms of duration and location of onset of PD (*P* > 0.05, Table 1). Among the 69 patients, nine were not taking anti-Parkinson's medication, six were taking levodopa combination therapy alone, one was taking a dopamine receptor agonist alone, and 53 were taking a combination of anti-Parkinson's drugs.

**Table 1:** Baseline characteristics of patients.

**Domain**	**PIGD (*n* = 34)**	**TD (*n* = 35)**	**HC (*n* = 38)**	***F/χ^2^/Z***	***P-*value**
**Sex**				4.528	0.104
Male	21 (61.8)	21 (60.0)	15 (39.5)		
Female	13 (38.2)	14 (40.0)	23 (60.5)		
Age (years)	66.59 ± 7.85	66.17 ± 6.39	67.34 ± 5.99	0.283	0.754
Duration (years)	4.00 (3.00, 8.25)	4.00 (2.00, 6.00)	–	−1.859	0.063
**Location of incidence**				0.018	0.894
Left	15 (44.1)	16 (45.7)	–		
Right	19 (55.9)	19 (54.3)	–		
Education (years)	9.00 (4.00, 12.00)	9.00 (6.00, 11.00)	8.00 (6.00, 10.50)	0.394	0.821
**Drugs**				2.316	0.678
Drug-free	5	4	–		
Dobasic levodopa compound preparations	3	3	–		
Dopamine receptor agonist	0	1	–		
Combination-drug	26	27	–		

### Characteristics of clinical symptoms in patients with PIGD, TD

**Figure 1:**
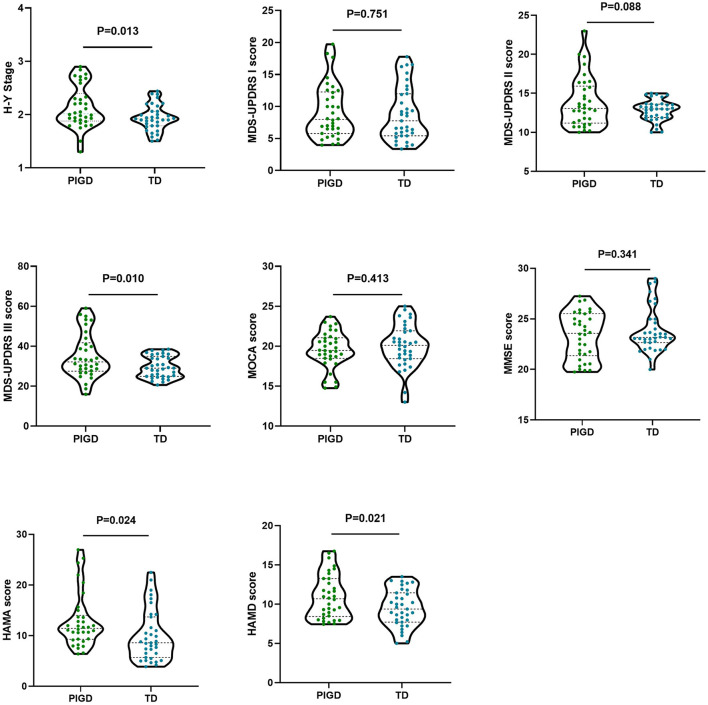
Characteristics of clinical symptoms in patients with PIGD, TD.

There were no significant differences between the two groups in MDS-UPDRS I (PIGD: 9.17 ± 4.21; TD: 8.84 ± 4.24), MDS-UPDRS II (PIGD: 13.84 ± 3.19; TD: 12.83 ± 1.33), MOCA (PIGD: 19.54 ± 2.29; TD: 20.04 ± 2.72), and MMSE scores (PIGD: 23.36 ± 2.31; TD: 23.94 ± 2.07; *P* > 0.05). The H-Y stage (PIGD: 2.07 ± 0.32; TD: 1.67 ± 0.23), MDS-UPDRS III scores (PIGD: 34.76 ± 10.56; TD: 27.44 ± 5.06), HAMA (PIGD: 12.88 ± 5.34; TD: 9.99 ± 5.06), and HAMD scores (PIGD: 10.95 ± 2.80; TD: 9.30 ± 2.23) were significantly higher than those in the TD group (*P* < 0.05, Figure 1).

### Characteristic parameters of different groups of VNG

The characteristic parameters of the VNG are analyzed in Table 2, and the left vertical sweep latencies were significantly different in the three groups (*P* = 0.047), with TD being the highest, followed by PIGD. The left vertical sweep latency phase was also significantly higher in PIGD than in HC (*P* = 0.049). Right vertical sweep accuracy (*P* = 0.019), 0.53 Hz SPEM horizontal gain (right; *P* = 0.004), 0.53 Hz SPEM horizontal gain (left; *P* = 0.008), 0.48 Hz SPEM vertical gain (up; *P* = 0.020), 0.8 Hz SPEM vertical gain (up; *P* = 0.000) and 0.8 Hz SPEM vertical gain (downward; *P* = 0.000) were significantly different in all three groups, with the PIGD group being the lowest, followed by TD.

**Table 2:** VNG characteristic parameters for PIGD, TD, and HC groups.

**VNG parameters**	**PIGD (*n* = 34)**	**TD (*n* = 35)**	**HC (*n* = 38)**	***F/χ^2^***	***P-*value**
Left horizontal sweep latency (ms)	229.94 ± 30.95	234.17 ± 25.52	229.32 ± 20.53	0.373	0.690
Right horizontal sweep latency (ms)	230.03 ± 23.72	233.23 ± 25.37	221.42 ± 21.49	2.477	0.089
Mean horizontal sweep latency (ms)	229.12 ± 22.26	233.37 ± 19.91	223.18 ± 18.51	2.345	0.101
Left horizontal sweep accuracy (%)	83 (72.75, 95)	80 (74, 86)	81.5 (75.75, 85.75)	0.567	0.753
Right horizontal sweep accuracy (%)	91 (78.75, 103)	88 (78, 96)	89.5 (84, 96.25)	0.723	0.697
Mean horizontal scanning accuracy (%)	88 (75.5, 95)	84 (78, 90)	86 (81, 91)	1.062	0.588
Left vertical sweep latency (ms)	245.5 (228.25, 258.25)	263 (234, 273)	239 (229.5, 258.75)^b^	6.120	**0.047** ^*^
Right vertical sweep latency (ms)	248.5 (226.75, 261)	239 (224, 258)	232 (219.75, 244)^a^	6.025	**0.049** ^*^
Mean vertical sweep latency (ms)	243.91 ± 18.75	245.83 ± 24.56	235.39 ± 18.42	2.643	0.076
Left vertical sweep accuracy (%)	66 (50.75, 81)	74 (62, 87)	74 (65, 86)	5.140	0.077
Right vertical sweep accuracy (%)	56.24 ± 19.64	62.34 ± 13.98	66.61 ± 11.58^a^	4.145	**0.019** ^*^
Mean vertical scanning accuracy (%)	63.32 ± 16.40	67.11 ± 12.01	70.13 ± 11.83	2.285	0.107
0.11 Hz SPEM level gain (right)	100.5 (82.75, 130)	102 (74, 116)	101 (90.75, 119.75)	0.434	0.805
0.11 Hz SPEM level gain (left)	105 (89.5, 128.5)	97 (77, 110)	102 (85, 113)	2.587	0.274
0.32 Hz SPEM level gain (right)	79 (60.75, 97.5)	93 (76, 101)	84.5 (66.5, 98)	1.947	0.378
0.32 Hz SPEM level gain (left)	84 (59, 98.5)	86 (76, 96)	86 (72.75, 98.25)	1.011	0.603
0.53 Hz SPEM level gain (right)	57 (35.5, 89.25)	79 (64, 94)^a^	88 (68.25, 99.25)^a^	10.960	**0.004** ^*^
0.53 Hz SPEM level gain (left)	63.5 (40.75, 83.5)	77 (51, 90)	86 (63.5, 97)^a^	9.704	**0.008** ^*^
0.16 Hz SPEM vertical gain (up)	67.5 (50, 87)	71 (54, 83)	70.5 (44.75, 93.25)	0.013	0.993
0.16 Hz SPEM vertical gain (down)	69 (53.75, 73.5)	72 (51, 80)	65.5 (41.5, 83.75)	0.393	0.822
0.48 Hz SPEM vertical gain (up)	43.47 ± 22.07	47.57 ± 23.50	58.53 ± 24.28^ab^	4.053	**0.020** ^*^
0.48 Hz SPEM vertical gain (down)	48.94 ± 22.05	54.31 ± 22.18	58.74 ± 22.98	1.713	0.185
0.8 Hz SPEM vertical gain (up)	21 (8.75, 32.75)	37 (18, 50)^a^	49.5 (30, 62.25)^ab^	20.156	**0.000** ^*^
0.8 Hz SPEM vertical gain (down)	24 (10, 35.25)	43 (19, 54)^a^	49.5 (32, 62.75)^ab^	21.475	**0.000** ^*^

^*^Indicates statistical significance at *p* < 0.05, and bold values highlight the variables with significant between-group differences. Superscript letters a and b indicate statistically significant pairwise differences in *post-hoc* comparisons. Values sharing the same letter are not significantly different, whereas values marked with different letters differ significantly between groups.

### Analysis of the correlation between MS and VNG

We analyzed the correlation between MDS-UPDRS III score and H-Y classification with VNG parameters in PIGD, TD patients. VNG in PIGD patients was negatively correlated with the MDS-UPDRS III score as evidenced by three parameters, namely, 0.32 Hz SPEM horizontal gain (right; *r* = −0.344, *P* = 0.036), 0.48 Hz SPEM vertical gain (up; *r* = −0.383, *P* = 0.025) and 0.8 Hz SPEM vertical gain (up; *r* = −0.390, *P* = 0.023). (*r* = −0.383, *P* = 0.025) and 0.8 Hz SPEM vertical gain (upward; *r* = −0.390, *P* = 0.023) parameters. There was no significant correlation between VNG and MDS-UPDRS III scores in TD patients (*P* > 0.05, Table 3).

**Table 3:** Correlation between MDS-UPDRS III score and VNG in PIGD, TD groups.

**VNG parameters**	**PIGD (*****n*** = **34)**		**TD (*****n*** = **35)**	
	***r***	***P-*** **value**	***r***	***P*** **-value**
Left horizontal sweep latency (ms)	−0.226	0.198	−0.129	0.462
Right horizontal sweep latency (ms)	0.124	0.484	0.003	0.988
Mean horizontal sweep latency (ms)	−0.015	0.931	−0.092	0.598
Left horizontal sweep accuracy (%)	−0.097	0.586	−0.146	0.402
Right horizontal sweep accuracy (%)	−0.162	0.359	−0.157	0.367
Mean horizontal scanning accuracy (%)	−0.161	0.364	−0.209	0.228
Left vertical sweep latency (ms)	−0.014	0.937	0.076	0.666
Right vertical sweep latency (ms)	−0.313	0.072	0.196	0.260
Mean vertical sweep latency (ms)	−0.183	0.301	0.119	0.495
Left vertical sweep accuracy (%)	−0.064	0.719	0.140	0.423
Right vertical sweep accuracy (%)	−0.031	0.863	−0.002	0.989
Mean vertical scanning accuracy (%)	−0.044	0.804	0.030	0.862
0.11 Hz SPEM level gain (right)	0.107	0.546	0.095	0.589
0.11 Hz SPEM level gain (left)	−0.147	0.405	−0.068	0.699
0.32 Hz SPEM level gain (right)	−0.344	**0.046** ^*^	−0.068	0.698
0.32 Hz SPEM level gain (left)	−0.315	0.070	0.285	0.097
0.53 Hz SPEM level gain (right)	−0.239	0.174	0.018	0.916
0.53 Hz SPEM level gain (left)	−0.303	0.082	−0.026	0.882
0.16 Hz SPEM vertical gain (up)	−0.103	0.560	−0.112	0.520
0.16 Hz SPEM vertical gain (down)	−0.112	0.530	−0.152	0.383
0.48 Hz SPEM vertical gain (up)	−0.383	**0.025** ^*^	−0.162	0.353
0.48 Hz SPEM vertical gain (down)	−0.323	0.063	−0.250	0.147
0.8 Hz SPEM vertical gain (up)	−0.390	**0.023** ^*^	−0.249	0.148
0.8 Hz SPEM vertical gain (down)	−0.129	0.462	−0.129	0.462

^*^Indicates statistical significance at *p* < 0.05, and bold values highlight the variables with significant between-group differences.

The severity of disease in patients with PIGD was negatively correlated with the VNG parameter, and the severity of disease in patients with TD was not correlated with the VNG (*P* > 0.05, Table 4). The correlation of PIGD was demonstrated in the left horizontal sweep latency (*r* = −0.391, *P* = 0.022), 0.32 Hz SPEM horizontal gain (left side; *r* = −0.354, *P* = 0.040), 0.8 Hz SPEM vertical gain (up; *r* = −0.372, *P* = 0.030), and 0.8 Hz SPEM vertical gain (down; *r* = −0.408, *P* = 0.017) were the four parameters.

**Table 4:** Correlation between H-Y staging and VNG in PIGD, TD groups.

**VNG parameters**	**PIGD (*****n*** = **34)**		**TD (*****n*** = **35)**	
	***r***	***P-*** **value**	***r***	***P-*** **value**
Left horizontal sweep latency (ms)	−0.391^*^	**0.022** ^*^	−0.055	0.755
Right horizontal sweep latency (ms)	−0.009	0.959	0.081	0.643
Mean horizontal sweep latency (ms)	−0.196	0.266	0.020	0.908
Left horizontal sweep accuracy (%)	−0.097	0.586	−0.188	0.279
Right horizontal sweep accuracy (%)	0.046	0.794	0.136	0.435
Mean horizontal scanning accuracy (%)	−0.042	0.814	0.017	0.923
Left vertical sweep latency (ms)	−0.210	0.234	0.174	0.318
Right vertical sweep latency (ms)	−0.331	0.056	0.000	0.999
Mean vertical sweep latency (ms)	−0.323	0.062	0.086	0.623
Left vertical sweep accuracy (%)	−0.258	0.141	0.302	0.078
Right vertical sweep accuracy (%)	0.094	0.598	0.029	0.871
Mean vertical scanning accuracy (%)	−0.007	0.971	0.180	0.301
0.11 Hz SPEM level gain (right)	0.268	0.125	−0.226	0.192
0.11 Hz SPEM level gain (left)	0.130	0.462	−0.111	0.525
0.32 Hz SPEM level gain (right)	−0.143	0.421	0.010	0.953
0.32 Hz SPEM level gain (left)	−0.354^*^	**0.040** ^*^	0.254	0.140
0.53 Hz SPEM level gain (right)	−0.094	0.599	−0.008	0.964
0.53 Hz SPEM level gain (left)	−0.293	0.092	−0.024	0.890
0.16 Hz SPEM vertical gain (up)	0.132	0.455	−0.012	0.947
0.16 Hz SPEM vertical gain (down)	0.049	0.782	−0.236	0.172
0.48 Hz SPEM vertical gain (up)	−0.303	0.082	−0.112	0.522
0.48 Hz SPEM vertical gain (down)	−0.280	0.109	−0.071	0.686
0.8 Hz SPEM vertical gain (up)	−0.372^*^	**0.030** ^*^	−0.063	0.720
0.8 Hz SPEM vertical gain (down)	−0.408^*^	**0.017** ^*^	−0.073	0.675

^*^Indicates statistical significance at *p* < 0.05, and bold values highlight the variables with significant between-group differences.

### Correlation analysis between NMS and VNG

The correlation between patients' mental status (HAMA, HAMD scores) and VNG was assessed. HAMA scores did not correlate with VNG in patients with PIGD, and HAMA scores were negatively correlated with VNG in patients with TD (Table 5). The correlation for TD was demonstrated in two parameters: left horizontal sweep accuracy (*r* = −0.384, *P* = 0.023), 0.16 Hz SPEM vertical gain (downward; *r* = −0.436, *P* = 0.009) parameters.

**Table 5:** Correlation between HAMA score and VNG in PIGD, TD groups.

**VNG parameters**	**PIGD (*****n*** = **34)**		**TD (*****n*** = **35)**	
	***r***	***P-*** **value**	***r***	***P-*** **value**
Left horizontal sweep latency (ms)	0.060	0.735	−0.063	0.718
Right horizontal sweep latency (ms)	0.236	0.179	−0.006	0.971
Mean horizontal sweep latency (ms)	0.229	0.192	−0.154	0.378
Left horizontal sweep accuracy (%)	−0.042	0.812	−0.384	**0.023** ^*^
Right horizontal sweep accuracy (%)	0.050	0.780	−0.101	0.564
Mean horizontal scanning accuracy (%)	0.058	0.745	−0.230	0.184
Left vertical sweep latency (ms)	0.244	0.165	0.016	0.929
Right vertical sweep latency (ms)	0.009	0.960	−0.042	0.809
Mean vertical sweep latency (ms)	0.167	0.346	−0.048	0.785
Left vertical sweep accuracy (%)	−0.017	0.925	0.136	0.436
Right vertical sweep accuracy (%)	−0.244	0.164	−0.003	0.988
Mean vertical scanning accuracy (%)	−0.064	0.720	0.069	0.695
0.11 Hz SPEM level gain (right)	−0.100	0.572	−0.146	0.403
0.11 Hz SPEM level gain (left)	−0.250	0.155	0.053	0.764
0.32 Hz SPEM level gain (right)	0.030	0.866	−0.226	0.191
0.32 Hz SPEM level gain (left)	−0.069	0.697	−0.231	0.182
0.53 Hz SPEM level gain (right)	−0.142	0.422	−0.029	0.868
0.53 Hz SPEM level gain (left)	−0.150	0.398	0.015	0.930
0.16 Hz SPEM vertical gain (up)	0.015	0.935	−0.093	0.597
0.16 Hz SPEM vertical gain (down)	−0.327	0.059	−0.436	**0.009** ^*^
0.48 Hz SPEM vertical gain (up)	−0.363^*^	0.035	0.080	0.646
0.48 Hz SPEM vertical gain (down)	−0.263	0.133	0.172	0.323
0.8 Hz SPEM vertical gain (up)	−0.273	0.119	0.043	0.804
0.8 Hz SPEM vertical gain (down)	−0.258	0.140	0.045	0.795

^*^Indicates statistical significance at *p* < 0.05, and bold values highlight the variables with significant between-group differences.

There were correlations between HAMD scores and VNG in both the PIGD and TD groups (Table 6). The correlation for PIGD was demonstrated by a positive correlation between HAMD scores and left vertical sweep latency (*r* = 0.405, *P* = 0.018), and positive correlations with right vertical sweep accuracy (*r* = −0.442, *P* = 0.009), 0.16 Hz SPEM vertical gain (downwards; *r* = −0.413, *P* = 0.015), and 0.48 Hz SPEM vertical gain (upward; *r* = −0.361, *P* = 0.036). The correlation for TD was demonstrated by HAMD scores that were negatively correlated with left side horizontal sweep accuracy (*r* = −0.501, *P* = 0.002), 0.16 Hz SPEM vertical gain (downward; *r* = −0.386, *P* = 0.022), and left side vertical sweep accuracy (*r* = −0.501, *P* = 0.002, *P* = 0.022) were negatively correlated.

**Table 6:** Correlation between HAMD score and VNG in PIGD, TD groups.

**VNG parameters**	**PIGD (*****n*** = **34)**		**TD (*****n*** = **35)**	
	***r***	***P-*** **value**	***r***	***P-*** **value**
Left horizontal sweep latency (ms)	0.185	0.295	−0.097	0.581
Right horizontal sweep latency (ms)	0.261	0.136	−0.035	0.844
Mean horizontal sweep latency (ms)	0.278	0.112	−0.157	0.369
Left horizontal sweep accuracy (%)	−0.114	0.521	−0.501^**^	**0.002** ^*^
Right horizontal sweep accuracy (%)	−0.101	0.570	−0.144	0.410
Mean horizontal scanning accuracy (%)	−0.135	0.447	−0.326	0.056
Left vertical sweep latency (ms)	0.405^*^	**0.018** ^*^	−0.080	0.648
Right vertical sweep latency (ms)	0.166	0.348	−0.116	0.505
Mean vertical sweep latency (ms)	0.313	0.072	−0.143	0.412
Left vertical sweep accuracy (%)	−0.141	0.427	0.077	0.661
Right vertical sweep accuracy (%)	−0.442^**^	**0.009** ^*^	−0.102	0.560
Mean vertical scanning accuracy (%)	−0.300	0.084	−0.033	0.849
0.11 Hz SPEM level gain (right)	−0.047	0.792	−0.237	0.170
0.11 Hz SPEM level gain (left)	−0.150	0.397	−0.047	0.790
0.32 Hz SPEM level gain (right)	−0.040	0.820	−0.194	0.264
0.32 Hz SPEM level gain (left)	−0.015	0.934	−0.124	0.478
0.53 Hz SPEM level gain (right)	−0.158	0.373	−0.066	0.705
0.53 Hz SPEM level gain (left)	−0.137	0.440	−0.001	0.997
0.16 Hz SPEM vertical gain (up)	−0.134	0.449	−0.032	0.857
0.16 Hz SPEM vertical gain (down)	−0.413^*^	**0.015** ^*^	−0.386^*^	**0.022** ^*^
0.48 Hz SPEM vertical gain (up)	−0.361^*^	**0.036** ^*^	0.006	0.972
0.48 Hz SPEM vertical gain (down)	−0.279	0.109	0.092	0.597
0.8 Hz SPEM vertical gain (up)	−0.168	0.342	−0.069	0.695
0.8 Hz SPEM vertical gain (down)	−0.112	0.528	−0.053	0.761

^*^Indicates *p* < 0.05 and ^**^ indicates *p* < 0.01, while bold values denote parameters showing statistically significant differences among the groups.

## Discussion

PD is a neurodegenerative disease with significant heterogeneity, which gradually accumulates the somatic motor system, autonomic nervous system and limbic system as the disease progresses. In this study, two subtypes of PD, PIGD, and TD, were investigated for the correlation between their clinical features and VNG. Statistically significant differences in VNG were found between the two groups, with PIGD patients having more severe disease severity (H–Y stage), motor symptoms (MDS-UPDRS III), and mental status (HAMA, HAMD) compared to TD patients. VNG was significantly correlated with clinical symptoms in PIGD patients, whereas in PD patients only mental status was correlated with VNG. This is the first comprehensive assessment of the correlation between VNG characteristics and clinical features in patients with PIGD and TD. A national study found differences in motoneural parameters between different populations by measuring eye movements in 49 patients with PD and 23 healthy people (23). Antoniades et al. also affirmed that motoneural function contributes to the diagnosis of PD, but requires the use of other indices to improve its specificity and sensitivity (24). However, the performance characteristics of eye movements in different subtypes of PD and the correlation with with other indicators have not been reported. Yang et al. (25) combined plasma and electroencephalographic markers, and the combination has correlation for the subtypes of PD, which provides a better means of identifying different subtypes (25). In order to increase the means of identification of PD subtypes, we used VNG to assess the oculomotor function of patients, combined with MS and NMS, to explore the correlation between these markers. There was no significant difference between the PIGD subtype and the TD subtype in the comparison of general data such as age, disease duration, and side of onset of disease in the enrolled PIGD subtype of this study, and the clinical features showed that the H-Y classification, MDS-UPDRS III, HAMA, and HAMD scores of the PIGD subtype were higher than those of the TD subtype, which further suggests that patients with the PIGD subtype have more severe MS and NMS than those with the TD subtype and the PIGD subtype has a more rapid rate of disease progression, which is consistent with the findings of Ren et al. faster rate of disease progression, which is consistent with the findings of Ren et al. (6). Greffard et al. (26) found that for every 1-point increase in UPDRS III, dopaminergic neurons were lost by 25/mm^3^, so the UPDRS III score is not only a basic tool for the assessment of motor symptoms in patients, but also can further provide a reference for exploring the pathophysiological mechanisms of PD. It is well-known that NMS is very common in PD patients, and Simuni et al. found that up to 88% of PD patients can exhibit various NMS (27). We found no significant difference in MMSE and MOCA scores between the two groups, PIGD and TD groups, which is consistent with the findings of Domellof et al. (28). This may be related to the duration of the disease and the H-Y classification of the subjects included in this study, which belonged to the early to middle stages of the disease and had not yet shown cognitive impairment. Studies have shown that 68%−88% of normal, age-equivalent individuals experience at least one type of NMS, but NMS tends to be more frequent and severe in patients with PD (29, 30). One study found that anxiety is present in about 40% of patients, while about 40%−50% of PD patients suffer from depression (31, 32). Thus, NMS is more disabling and has a greater impact on the quality of life of PD patients compared to MS (33). One study showed that depression, anxiety, and sleep symptoms were more severe in patients with the new-onset PIGD subtype compared with those with the TD subtype, and that depressive symptoms were an independent risk factor for the PIGD subtype (34), which is consistent with the results of the present study.

Examining eye movements is an important part of the neurological examination, and most patients with neurodegenerative diseases are often associated with different forms and degrees of eye movement abnormalities (21, 35). Increasingly, oculomotor function tests are now available to assess the functional integrity of brain systems involved in sensorimotor activity (36, 37). Abnormalities in oculomotor function can often provide clues to localize disease damage if they may be associated with specific pathophysiological or anatomical structural abnormalities in the brain (38). We compared the oculomotor parameters of the PIGD subtype group, the TD subtype group, and the HC group, and found that the left horizontal sweep latency, the right horizontal sweep latency, the mean horizontal sweep latency, the mean vertical sweep latency, the 0.11 Hz SPEM horizontal gain, the 0.11 Hz SPEM horizontal gain (left side), and the 0.32 Hz SPEM horizontal gain (right side), 0.32 Hz SPEM horizontal gain (left side), 0.16 Hz SPEM vertical gain (up), 0.16 Hz SPEM vertical gain (down), and 0.48 Hz SPEM vertical gain (down) were not significantly different. This is inconsistent with previous findings, in which reduced sweep amplitude is detected early in the course of the disease in patients with PD, which may reflect degeneration of the basal ganglia (15). We speculate that it may be related to the examination method, equipment instrumentation, individualization of patients, or perhaps the vast majority of subjects were in the early to middle stages of the disease and failed to reflect significant intergroup differences. Whereas, TD subtype, PIGD subtype and HC compared to left vertical scanning latency, right vertical scanning latency, 0.53 Hz SPEM horizontal gain (right), 0.53 Hz SPEM horizontal gain (left), 0.48 Hz SPEM vertical gain (up), 0.8 Hz SPEM vertical gain (up), 0.8 Hz SPEM vertical gain (down) had a significant difference, we found that there was asymmetry in smooth tracking motor gain and the higher the SPEM frequency, the greater the significance of the difference, while the PIGD subtype had a lower SPEM gain than the TD subtype. Zhang et al. (23) found that vertical scanning abnormalities were associated with disease progression in PD, and we found in this study that the vertical scanning abnormalities in the PIGD subtype were more pronounced than in the TD subtype, which might correlate with disease progression among PD patients with different motor subtypes.

The results of the VNG were further correlated with the clinical features and significant negative correlations were found between the H–Y grading of the PIGD subtype and the left horizontal sweep latency, 0.32 Hz SPEM horizontal gain (left), 0.8 Hz SPEM vertical gain (up), and 0.8 Hz SPEM vertical gain (down). There was a significant negative correlation between anxiety and both 0.48 Hz SPEM vertical gain (up). Significant negative correlations were found between depression and right vertical sweep accuracy, 0.16 Hz SPEM vertical gain (downward), 0.48 Hz SPEM vertical gain (upward), and significant positive correlations with left vertical sweep latency. Significant negative correlations were found between MDS-UPDRS III and 0.32 Hz SPEM horizontal gain (right), 0.48 Hz SPEM vertical gain (upward), and 0.8 Hz SPEM vertical gain (upward), while there were significant negative correlations between anxiety, depression, and left-sided horizontal scanning accuracy, and 0.16 Hz SPEM vertical gain (downward) for the TD subtype. Carey et al. (39) found that anxiety symptoms were associated with altered limbic cortex-striatal thalamocortical circuits through a study of neuroimaging, whereas Depressive symptoms correlated with dopaminergic reductions in the striatum, amygdala, prefrontal, temporal and parietal regions, suggesting a significant correlation between the development of depression and anxiety and hypoplasia of the striatal pathway (39), as well as suggesting that the clinical features of the PIGD subtype affect the oculomotor condition more significantly than the TD subtype. However, the correlation coefficients in the reported results were all below 0.5, indicating that the correlations were moderately weak. In complex neurological disorders such as PD, where both motor and non-motor symptoms stem from distributed network dysfunction, small effect sizes are not uncommon. Nevertheless, even weak correlations may hold biological significance, particularly when they align with existing pathophysiological models (40, 41).

Overall, the differential presentation of VNG parameters in patients with the TD/PIGD subtype suggests that these indicators may serve as potential biomarkers for distinguishing between PD subtypes. This finding also reveals possible differences in neural circuitry or central integration functions across subtypes. Furthermore, investigating the correlation between VNG parameters and non-motor symptoms in TD/PIGD subtype patients aids in understanding vestibular dysfunction variations across subtypes. This may provide a basis for risk assessment and early intervention in distinct PD subtypes, demonstrating clinical utility. Consequently, this study adheres to the MSD guidelines.

## Conclusion

VNG parameters for the PIGD subtype correlate with both MS and NMS, whereas VMG parameters for the TD subtype correlate exclusively with NMS. This indicates that VNG parameters hold potential for distinguishing intrinsic differences between PD motor subtypes, and that these parameters exhibit variability across non-motor symptoms in patients with different subtypes, thereby deepening our understanding of PD heterogeneity.

## Limitations

This study retains certain limitations. Firstly, this single-center study involved a relatively small patient cohort and lacked individuals with atypical intermediate-type symptoms, resulting in a bias toward the absence of intermediate-type patients within our cohort. Secondly, most enrolled PD patients were in the early-to-mid stages of the disease, and the absence of longitudinal follow-up means conclusions cannot be extrapolated to patients across all disease stages. Finally, this study failed to exclude the influence of medication on ocular motility. The vast majority of patients had received anti-PD medication prior to examination, which may have impacted the findings. Therefore, it is recommended that multicentre, multi-sample, longitudinal, data-driven studies be employed to optimize the quality ratings of PD subtype research (42).

## Data Availability

The datasets presented in this study can be found in online repositories. The names of the repository/repositories and accession number(s) can be found in the article/Supplementary material.

## References

[1] Zhang ZX Roman GC Hong Z Wu CB Qu QM Huang JB . Parkinson's disease in China: prevalence in Beijing, Xian, and Shanghai. Lancet. (2005) 365:595–7. doi: 10.1016/S0140-6736(05)70801-1 15708103

[2] Munhoz RP Tumas V Pedroso JL Silveira-Moriyama L. The clinical diagnosis of Parkinson's disease. Arq Neuropsiquiatr. (2024) 82:1–10. doi: 10.1055/s-0043-1777775 PMC10849824 38325391

[3] Leite Silva ABR Gonçalves de Oliveira RW Diógenes GP de Castro Aguiar MF Sallem CC Lima MPP . Premotor, nonmotor and motor symptoms of Parkinson's Disease: a new clinical state of the art. Ageing Res Rev. (2023) 84:101834. doi: 10.1016/j.arr.2022.101834 36581178

[4] Thenganatt MA Jankovic J. Parkinson disease subtypes. JAMA Neurol. (2014) 71:499–504. doi: 10.1001/jamaneurol.2013.6233 24514863

[5] Choi SM Kim BC Cho BH Kang KW Choi KH Kim JT . Comparison of two motor subtype classifications in *de novo* Parkinson's disease. Parkinsonism Relat Disord. (2018) 54:74–8. doi: 10.1016/j.parkreldis.2018.04.021 29703644

[6] Ren J Hua P Li Y Pan C Yan L Yu C . Comparison of three motor subtype classifications in *de novo* Parkinson's disease patients. Front Neurol. (2020) 11:601225. doi: 10.3389/fneur.2020.601225 33424750 PMC7785849

[7] Gallea C Wicki B Ewenczyk C Rivaud-Péchoux S Yahia-Cherif L Pouget P . Antisaccade, a predictive marker for freezing of gait in Parkinson's disease and gait/gaze network connectivity. Brain. (2021) 144:504–14. doi: 10.1093/brain/awaa407 33279957

[8] White OB Saint-Cyr JA Tomlinson RD. Ocular motor deficits in Parkinson's disease. II Control of the saccadic and smooth pursuit systems. Brain. (1983) 106:571–87. doi: 10.1093/brain/106.3.571 6640270

[9] Wu L Wang Q Zhao L Jiang CY Xu Q Wu SC . Clinical and oculomotor correlates with freezing of gait in a chinese cohort of Parkinson's disease patients. Front Aging Neurosci. (2020) 12:237. doi: 10.3389/fnagi.2020.00237 32903684 PMC7438737

[10] Ewenczyk C Mesmoudi S Gallea C. Antisaccades in Parkinson disease: a new marker of postural control? Neurology. (2017) 88:853–61. doi: 10.1212/WNL.0000000000003658 28130466

[11] Schlenstedt C Muthuraman M Witt K Weisser B Fasano A Deuschl G. Postural control and freezing of gait in Parkinson's disease. Parkinsonism Relat Disord. (2016) 24:107–12. doi: 10.1016/j.parkreldis.2015.12.011 26762797

[12] Wang J Qiao Z Cao X Li H Wang Y Jiao Q . G Protein-coupled receptors: key targets for maintaining the function of basal ganglia-thalamus-cortical circuits in Parkinson's disease. Biochem Pharmacol. (2025) 242(Pt 2):117303. doi: 10.1016/j.bcp.2025.117303 40912368

[13] Gibson JM Pimlott R Kennard C. Ocular motor and manual tracking in Parkinson's disease and the effect of treatment. J Neurol Neurosurg Psychiatry. (1987) 50:853–60. doi: 10.1136/jnnp.50.7.853 3625208 PMC1032122

[14] Lal V Truong D. Eye movement abnormalities in movement disorders. Clin Park Relat Disord. (2019) 1:54–63. doi: 10.1016/j.prdoa.2019.08.004 34316601 PMC8288550

[15] Anderson TJ MacAskill MR. Eye movements in patients with neurodegenerative disorders. Nat Rev Neurol. (2013) 9:74–85. doi: 10.1038/nrneurol.2012.273 23338283

[16] Pinkhardt EH Kassubek J. Ocular motor abnormalities in Parkinsonian syndromes. Parkinsonism Relat Disord. (2011) 17:223–30. doi: 10.1016/j.parkreldis.2010.08.004 20801069

[17] Bronstein AM Kennard C. Predictive ocular motor control in Parkinson's disease. Zhonghua Yi Xue Za Zhi. (2008) 88:442–4. 18642781

[18] Zeigelboim BS Teive HAG Sampaio R. Electronystagmography findings in spinocerebellar ataxia type 3 (SCA3) and type 2 (SCA2). Arq Neuropsiquiatr. (2011) 69:760–5. doi: 10.1590/S0004-282X2011000600007 22042177

[19] Marras C Fereshtehnejad SM Berg D Bohnen NI Dujardin K Erro R . Transitioning from subtyping to precision medicine in Parkinson's disease: a purpose-driven approach. Mov Disord. (2024) 39:462–71. doi: 10.1002/mds.29708 38243775

[20] Chung CL Mak MK Hallett M. Transcranial magnetic stimulation promotes gait training in Parkinson disease. Ann Neurol. (2020) 88:933–45. doi: 10.1002/ana.25881 32827221 PMC8470277

[21] Sun YR Beylergil SB Gupta P Ghasia FF Shaikh AG. Monitoring eye movement in patients with Parkinson's disease: what can it tell us? Eye Brain. (2023) 15:101–12. doi: 10.2147/EB.S384763 37519412 PMC10377572

[22] Beh SC Frohman TC Frohman EM. Cerebellar control of eye movements. J Neuroophthalmol. (2017) 37:87–98. doi: 10.1097/WNO.0000000000000456 27643747

[23] Zhang J Zhang B Ren Q Zhong Q Li Y Liu G . Eye movement especially vertical oculomotor impairment as an aid to assess Parkinson's disease. Neurol Sci. (2021) 42:2337–45. doi: 10.1007/s10072-020-04796-6 33043395

[24] Antoniades CA Spering M. Eye movements in Parkinson's disease: from neurophysiological mechanisms to diagnostic tools. Trends Neurosci. (2024) 47:71–83. doi: 10.1016/j.tins.2023.11.001 38042680

[25] Yang X Li Z Bai L Shen X Wang F Han X . Association of plasma and electroencephalography markers with motor subtypes of Parkinson's disease. Front Aging Neurosci. (2022) 14:911221. doi: 10.3389/fnagi.2022.911221 35903537 PMC9314775

[26] Greffard S Verny M Bonnet AM Beinis JY Gallinari C Meaume S . Motor score of the unified Parkinson disease rating scale as a good predictor of Lewy body-associated neuronal loss in the substantia nigra. Arch Neurol. (2006) 63:584–8. doi: 10.1001/archneur.63.4.584 16606773

[27] Simuni T Sethi K. Nonmotor manifestations of Parkinson's disease. Ann Neurol. (2008) 64(Suppl 2):S65–80. doi: 10.1002/ana.21472 19127582

[28] Domellof ME Elgh E Forsgren L. The relation between cognition and motor dysfunction in drug-naive newly diagnosed patients with Parkinson's disease. Mov Disord. (2011) 26:2183–9. doi: 10.1002/mds.23814 21661051

[29] Shamim EA Chu J Scheider LH Savitt J Jinnah HA Hallett M. Extreme task specificity in writer's cramp. Mov Disord. (2011) 26:2107–9. doi: 10.1002/mds.23827 21714006 PMC3417074

[30] Khoo TK Yarnall AJ Duncan GW Coleman S O'Brien JT Brooks DJ . The spectrum of nonmotor symptoms in early Parkinson disease. Neurology. (2013) 80:276–81. doi: 10.1212/WNL.0b013e31827deb74 23319473 PMC3589180

[31] Pontone GM Williams JR Anderson KE Chase G Goldstein SA Grill S . Prevalence of anxiety disorders and anxiety subtypes in patients with Parkinson's disease. Mov Disord. (2009) 24:1333–8. doi: 10.1002/mds.22611 19425086 PMC2830642

[32] Reijnders JS Ehrt U Weber WE Aarsland D Leentjens AF. A systematic review of prevalence studies of depression in Parkinson's disease. Mov Disord. (2008) 23:183–9. doi: 10.1002/mds.21803 17987654

[33] Pfeiffer RF. Non-motor symptoms in Parkinson's disease. Parkinsonism Relat Disord. (2016) 22(Suppl 1):S119–22. doi: 10.1016/j.parkreldis.2015.09.004 26372623

[34] Ren J Hua P Pan C Li Y Zhang L Zhang W . Non-motor symptoms of the postural instability and gait difficulty subtype in *de novo* Parkinson's disease patients: a cross-sectional study in a single center. Neuropsychiatr Dis Treat. (2020) 16:2605–12. doi: 10.2147/NDT.S280960 33173298 PMC7646450

[35] Frei K. Abnormalities of smooth pursuit in Parkinson's disease: a systematic review. Clin Park Relat Disord. (2021) 4:100085. doi: 10.1016/j.prdoa.2020.100085 34316663 PMC8299966

[36] Larrazabal AJ Cena CEG Martinez CE. Video-oculography eye tracking towards clinical applications: a review. Comput Biol Med. (2019) 108:57–66. doi: 10.1016/j.compbiomed.2019.03.025 31003180

[37] Fooken J Patel P Jones CB McKeown MJ Spering M. Preservation of eye movements in Parkinson's disease is stimulus- and task-specific. J Neurosci. (2022) 42:487–99. doi: 10.1523/JNEUROSCI.1690-21.2021 34848498 PMC8802919

[38] Gorges M Maier MN Rosskopf J Vintonyak O Pinkhardt EH Ludolph AC . Regional microstructural damage and patterns of eye movement impairment: a DTI and video-oculography study in neurodegenerative parkinsonian syndromes. J Neurol. (2017) 264:1919–28. doi: 10.1007/s00415-017-8579-8 28762086

[39] Carey G Görmezoglu M de Jong JJA Hofman PAM Backes WH Dujardin K . Neuroimaging of anxiety in Parkinson's disease: a systematic review. Mov Disord. (2021) 36:327–39. doi: 10.1002/mds.28404 33289195 PMC7984351

[40] Parkinson Progression Marker Initiative. The Parkinson Progression Marker Initiative (PPMI). Prog Neurobiol. (2011) 95:629–35. doi: 10.1016/j.pneurobio.2011.09.005 21930184 PMC9014725

[41] Obeso JA Rodríguez-Oroz MC Benitez-Temino B Blesa FJ Guridi J Marin C . Functional organization of the basal ganglia: therapeutic implications for Parkinson's disease. Mov Disord. (2008) 23(Suppl 3):S548–59. doi: 10.1002/mds.22062 18781672

[42] Mestre TA Fereshtehnejad SM Berg D Bohnen NI Dujardin K Erro R . Parkinson's disease subtypes: critical appraisal and recommendations. J Parkinsons Dis. (2021) 11:395–404. doi: 10.3233/JPD-202472 33682731 PMC8150501

