# Supplementary material for: Videonystagmography features and clinical symptoms correlate with Parkinson's clinical subtypes
Source: Front Neurol. 2025 Nov 26;16:1693803. doi: 10.3389/fneur.2025.1693803 (PMC12689390; doi:10.3389/fneur.2025.1693803)
Supplement: Supplementary file 1 [file Table_1.docx]

**VNG data acquisition and preprocessing**

The ZT-VNG-I Benign Paroxysmal Positional Vertigo Diagnostic Instrument manufactured by Tianjin Zhihearing Medical Technology Co., Ltd. in the inpatient department of Neurology was selected, with a screen size of L62*W110 and a screen resolution of 1920*1080. The eye movements of the subjects were examined, and the test was carried out in a quiet and dark environment, and the eye movements of all the subjects were evaluated at the bedside before the test task. The eye movements were assessed at the bedside by asking the subjects to follow the examiner's finger or a pencil at a distance of approximately 1 m. The subjects were asked not to move their head when instructed not to do so. Subjects should be able to gaze at the target when instructed not to move their head. The speed, direction, amplitude and smoothness of the eye movements were observed. Subjects were then instructed to adapt to a dark environment for 5 min and to practice beforehand. Subjects were asked to sit with their head fixed 120 cm from the TV screen, and were instructed to concentrate on the white dot cursor on the video screen to correct the video signal and to ensure that the camera was able to accurately capture the eye movements before the test could be formally started. Eye movement trajectories were recorded in the horizontal plane to assess the eye movement task. When examining eye movements, attention was paid to the deviation of eye movements from the desired gaze direction to ensure quality. A video eyepiece equipped with an infrared camera is worn on the head to determine the centre of the pupil, and the camera lens will take real-time recordings of the changes in the movement position produced by the eyeballs, and then the recorded electrical signals of the amount of change in the movement position produced by the eyeballs will be transmitted to a computer that is connected to itself, and the computer will identify the characteristics of the VNG waveforms and process them, and display the waveforms of the VNG on the monitor, and based on the At the same time, based on the amount of movement position change of the eyeball, finally the computer will calculate the movement parameters such as the latency of the eyeball movement sweep, the accuracy of the sweep, and the value of the smooth tracking gain at different frequencies, and the movement parameter data will be used as a diagnostic reference. The observed motor parameter indexes mainly included the latency of the sweep, sweep accuracy, and smooth tracking gain: ① Horizontal and vertical visually-mediated reflexive sweeps: the purpose of the fixation was to measure the relationship between the eye displacements at a certain viewing angle and the corresponding recorded signals (oculomotor curve displacements), which were used for the calculation of parameters such as oculomotor amplitude and velocity. The starting position of the reticle was at the centre (0°), and then the subject alternated between looking forward and centrally at the reticle with a 20° left-right paracentral and a 10° up-down paracentral angle at intervals of >1 s. The subject was asked to look at the reticle with the eye at the centre and the reticle with the eye at the centre and the reticle at the centre. Subjects were required to stare at the visual field and move their eyes rapidly and accurately following the jumps of the visual field in different directions, and the latency and accuracy of the sweep were recorded. Sweep latency is the time difference between eye movement and the movement of the visual target, and sweep accuracy is the ratio of eye amplitude to the amplitude of the visual target × 100%. ②SPEM: Subjects should always follow the reticle on the screen, which moves regularly back and forth on the horizontal and vertical axes, left and right, and up and down, respectively, and record the eye movements when the reticle oscillates sinusoidally at six frequencies: 0.11 Hz, 0.32 Hz, 0.53 Hz, 0.16 Hz, 0.48 Hz, 0.8 Hz, etc.; 0.11 Hz, 0.32 Hz, 0.53 Hz, 0.48 Hz, 0.48 Hz, 0.8 Hz, 0.48 Hz, 0.48 Hz, 0.48 Hz, 0.48 Hz, 0.48 Hz, 0.48 Hz, 0.53 Hz, 0.16 Hz, 0.48 Hz, and 0.8 Hz, the SPEM corresponds to the optic target spot moving at 10°/s, 30°/s, and 50°/s, respectively. Recordings were made by the VNG analyser system, which calculated the subject's SPEM gain, scanning latency, and scanning accuracy, and then the results were examined by the testing physician to remove any inadvertent blinking, head rotation, and measurement errors. The test results were then checked by the testing physician to remove the eye movement data due to blinking, inadvertent head rotation and measurement errors so as not to interfere with the statistical results. All VNG examinations were performed by two neurologists with specialised training in VNG and Parkinson's disease.
